# Repeated measures of PAP at different elevations in beef bulls in Colorado^1,2^

**DOI:** 10.1093/tas/txaa116

**Published:** 2020-12-22

**Authors:** Taylor R Zimprich, Scott E Speidel, David W Schafer, Beth Lashell, Timothy N Holt, R Mark Enns, Sam F Cunningham, Milton G Thomas

**Affiliations:** 1 Department of Animal Science, Colorado State University, Fort Collins, CO; 2 Colorado Agricultural Experiment Station, Colorado State University, Fort Collins, CO; 3 Department of Agriculture and Biology, Fort Lewis College, Durango, CO; 4 Department of Clinical Sciences, Colorado State University, Fort Collins, CO

## INTRODUCTION

Pulmonary arterial pressure (PAP) is a measure for pulmonary hypertension and to determine an individual’s susceptibility to high mountain disease (HMD). Right-sided heart failure that results from the hypoxic conditions at altitudes greater than 1,500 m is a common pathophysiological observation of HMD (Thomas et al., 2018). Past research indicates that PAP measurements are moderately heritable, which is important when selecting bulls for high-altitude beef production systems (Shirley et al., 2008; Crawford et al., 2016; Pauling et al., 2018). Bull and heifer development procedures for these systems typically PAP test cattle at approximately 1 yr of age (culling of animals with measurements >50 mmHg; BIF, 2020). Yearling PAP expected progeny differences (EPD) are now being published by several breeding organizations; therefore, there is need to learn more about this trait and the many sources of variation (breed, age, altitude, etc.) that may influence breeding value estimations.

This project’s overarching goals were to determine whether a bull’s lifetime PAP can be determined by a yearling measurement at moderate elevation. We are also determining the impact of changing altitude on a bull’s PAP measurements and the relationship between PAP at moderate altitude (1,525 m) to PAP at high altitude (2,470 m). With EPD for PAP measurement becoming available within breed associations such as American Angus Association (Angus Genetics Inc., 2019), it is important to determine the implications of low to moderate elevation PAP measurements on overall usefulness in estimating high-altitude PAP EPD and for the movement of bulls to/from moderate and high elevations. Due to the aforementioned, the objective of this study was to analyze repeated measures of PAP in growing beef bulls and determine correlations across time elevation.

## MATERIALS AND METHODS

This study obtained approval from the Colorado State University Animal Care and Use Committee under protocol number 16-6757AA.

### Cattle and Data

Pulmonary arterial pressure was measured on Angus and Hereford bulls (*n* = 18 and *n* = 12, respectively) at different ages and elevations as the bulls progressed from weaning to yearling to 18 mo of age. Cattle were born at an elevation of approximately 1,525 m located at Colorado State University’s Agricultural Research, Development, and Education Center (ARDEC) facility located in Fort Collins, CO. Bulls used in this study were calved in the spring months ranging from March to May 2018. Post-weaning, bulls were fed a typical gain test ration (1.5 kg/d). Subsequently, bulls were moved and grazed irrigated pasture, gaining ~0.5 kg/d at Fort Lewis College (Hesperus, CO) at an elevation of approximately 2,470 m from June to September 2019. The bulls then returned to ARDEC on 11 September 2019. Five PAP measurements were collected from each bull over this time period: 1) weaning PAP at ARDEC, 2) yearling PAP at ARDEC, 3) after acclimating to high altitude (FLC), 4) before returning to ARDEC from FLC, and 5) after acclimating again to the moderate altitude at ARDEC. Due to these data collection procedures, elevation and PAP (collection date) were confounded.

### Statistical Analysis

Model selection was completed using stepwise regression to determine the most important terms to be included in the model. The model utilized was as follows:

$$\begin{array}{l} y_{i}=X_{i}b+Z_{i}u+e_{i} \end{array}$$

where ***y***_***i***_ was a vector of observations referring to the trait *i.****X***_***i***_ was an incidence matrix relating the fixed effects in vector ***b***_***i***_ to the observations ***y***_***i***_. In addition, the effects of a quadratic random regression of PAP on time within an individual were included as incidence matrix ***Z***_***i***_, relating the values of the PAP regression to the observations in ***y***_***i***_. The value ***e***_***i***_ corresponded to the residual errors associated with the vector of observations.

The data were then analyzed using the statistical software package R and ASReml 3.0 (Gilmour et al., 2009) to test differences in PAP within individuals at different ages and elevations. This was used to determine predicted PAP values. Spearman rank correlations and Pearson correlations for PAP on time (elevation/age confounding) were then evaluated from the fitted values for the regression line.

## RESULTS AND DISCUSSION

**Figure 1.**
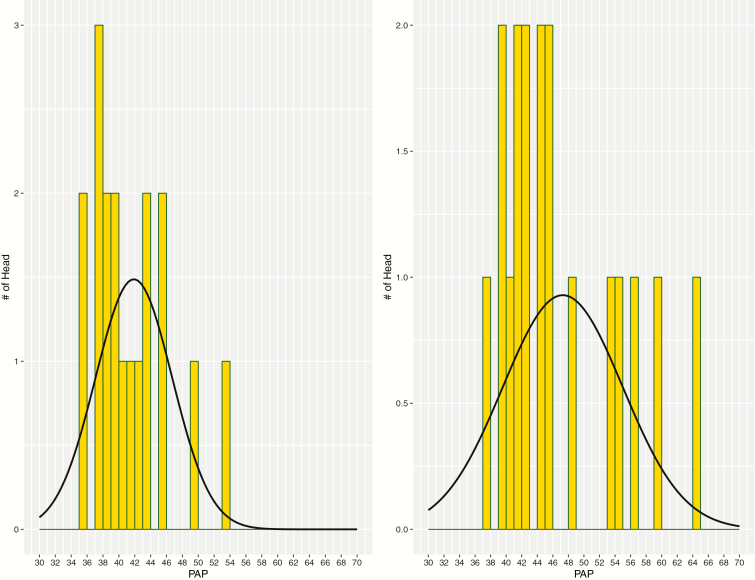
Variation among Angus bulls between time 2 (yearling PAP at elevation = 1525 m) and time 3 (age = 14.0 ± 0.1 mo; elevation = 2,470 m).

**Figure 2.**
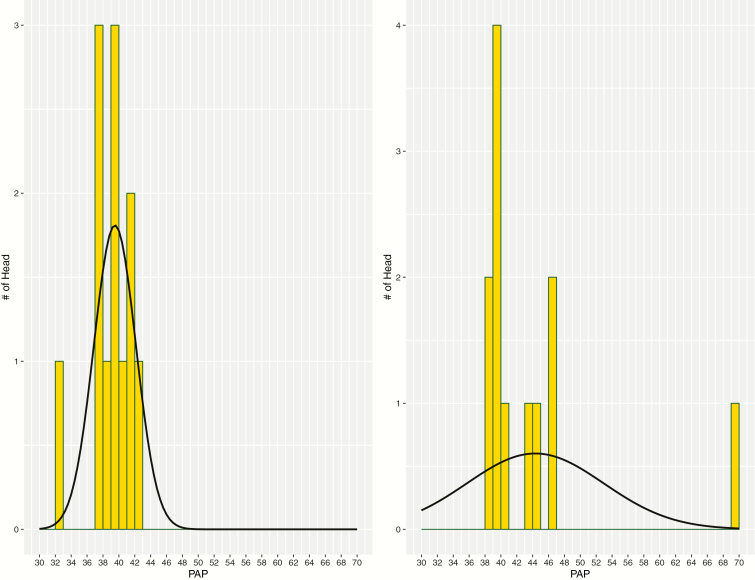
Variation among Hereford bulls between time 2 (yearling PAP at elevation = 1,525 m) and time 3 (age = 14.0 ± 0.1 mo; elevation = 2,470 m).

Number of observations, arithmetic PAP means, PAP SD, elevation, ages, body weight, as well as minimum and maximum values for PAP in both Black Angus bulls and Hereford bulls are presented in Tables 1 and 2, respectively. Note the increase in PAP mean and SD as bulls aged and were exposed to higher elevation and then moved back to moderate elevation. This increase in PAP variability can be observed in Figs. 1 and 2. These two figures contain the distribution of PAP observations between times 2 and 3 for Angus (Fig. 1) and Hereford (Fig. 2). Also observed, this variation decreases after bulls are at elevation for a longer period of time and then return to moderate elevation. These changes in variation of PAP with age were also observed in study of Neary et al. (2015). Overall, Neary et al. (2015) suggests that as cattle age and are exposed to high elevation hypoxic environments, the number of extreme high PAP individual observations also increases. This expansion of the right-side of a distribution curve has been documented in previous studies of Angus cattle and PAP (Pauling et al., 2018; Cockrum et al., 2019).

**Table 1.** Summary Statistics of Angus Bulls (arithmetic means)

Time	Location	Average age (d) ± SE	Average weight (kg) ± SE	*N*	Mean	SD	Min	Max
1	ARDEC	185 ± 3.9	247.9 ± 5.2	18	38.11	2.49	34	44
2	ARDEC	369 ± 3.9	555.1 ± 9.1	18	41.89	4.83	36	54
3	FLC	424 ± 3.9	—	18	47.28	7.73	38	65
4	FLC	500 ± 3.9	568.8 ± 8.9	18	45.67	6.22	37	57
5	ARDEC	553 ± 3.9	671.5 ± 9.5	18	42.39	2.95	37	49

ARDEC = 1,525 m; FLC = 2,470 m.

**Table 2.** Summary statistics of Hereford bulls (arithmetic means)

Time	Location	Average age (d) ± SE	Average weight (kg) ± SE	*N*	Mean	SD	Min	Max
1	ARDEC	195 ± 3.9	239.2 ± 9.4	12	37.75	1.06	36	39
2	ARDEC	379 ± 3.9	542.1 ± 11.4	12	39.50	2.65	33	43
3	FLC	434 ± 3.9	—	12	44.33	8.62	39	70
4	FLC	510 ± 3.9	561.0 ± 11.3	12	44.00	5.91	39	60
5	ARDEC	563 ± 3.9	650.8 ± 13.8	12	42.00	4.86	37	54

ARDEC = 1,525 m; FLC = 2,470 m.

PAP and body weight differed (*P* < 0.05) among the five time points. PAP also differed (*P* < 0.05) among the breeds at these five times. Means of each breed pooled across these times were 41.2 ± 1.3 (Hereford) < 43.2 ± 1.4 (Angus). Pearson correlations (above diagonal) and Spearman’s rank correlation (below diagonal) between the fitted values from the regression model are shown in Table 3. Correlations between yearling PAP (time 2) and PAP measured at a higher elevation times 3 and 4 were highly correlated. The correlations between yearling PAP (2) and PAP at high elevation (2 and 3) were 0.99 and 0.93, respectively. Interestingly, the correlations between the high elevation PAPs and PAP after acclimating again to moderate elevation (time 5) were moderate. The correlation between initial PAP at high altitude (3) and after acclimation (5) was 0.60. The second high elevation PAP (4) was more correlated with after acclimation (5) at 0.77. Historically, weaning PAP has been considered to be less accurate than yearling PAP (Holt and Callan, 2007; Zeng et al., 2016); this study also supports these findings. The rank correlations presented in the current study yielded similar results. Overall, this study provided evidence to help understand how yearling PAP will relate to measures later in life and at differing elevations. However, we must clarify that this study first measured bulls at 1,525 m, then at 2,470 m, and then again at 1,525 m. Pauling et al. (2018) reported that 1,520 m was the inflection point where altitude starts to impact hypoxia-induced increases in PAP. Therefore, the altitude of the ARDEC facility of CSU may be high enough to determine whether a bull has PAP that will be acceptable or unacceptable for mountainous beef production systems, yet this warrants additional research. The current study also provided additional results of the influences of age-growth on PAP measures. Neary et al. (2015) reported PAP increased with age and weight. This study involved changes in altitude; therefore, altitude, specifically high altitude, yielded increased PAP measurements at those time points (4 and 5).

**Table 3.** Pearson correlations (above diagonal) and Spearman’s rank correlations (below diagonal) of predictions for PAP indication of additional PAP measurements

	1^A^	2^A^	3^B^	4^B^	5^A^
1^A^		0.62	0.51	0.50	0.42
2^A^	0.55		0.99	0.93	0.97
3^B^	0.48	0.98		0.97	0.60
4^B^	0.43	0.92	0.97		0.77
5^A^	0.42	0.63	0.71	0.82	

A = ARDEC (1,525 m); B = FLC (2,470 m).

## IMPLICATIONS

This study suggested that yearling PAP was the best estimate of future PAP performance in beef bulls. Therefore, these data support the initial research finding that were used in the development of the American Angus Association PAP EPD, which defines the EPD as being a trait of yearling PAP for cattle of high altitude (1,520 m).
